# Altered resting‐state functional connectivity of the putamen and internal globus pallidus is related to speech impairment in Parkinson's disease

**DOI:** 10.1002/brb3.1073

**Published:** 2018-07-25

**Authors:** Jordan L. Manes, Kris Tjaden, Todd Parrish, Tanya Simuni, Angela Roberts, Jeremy D. Greenlee, Daniel M. Corcos, Ajay S. Kurani

**Affiliations:** ^1^ Department of Physical Therapy and Human Movement Sciences Northwestern University Chicago Illinois; ^2^ Department of Communication Disorders and Sciences University at Buffalo Buffalo New York; ^3^ Department of Radiology Northwestern University Chicago Illinois; ^4^ Ken and Ruth Davee Department of Neurology Northwestern University Chicago Illinois; ^5^ The Parkinson's Disease and Movement Disorders Clinic Northwestern University Chicago Illinois; ^6^ Roxelyn and Richard Pepper Department of Communication Sciences and Disorders Northwestern University Evanston Illinois; ^7^ Department of Neurosurgery University of Iowa Iowa City Iowa

**Keywords:** basal ganglia, dysarthria, fMRI, Parkinson's disease, speech disorders

## Abstract

**Introduction:**

Speech impairment in Parkinson's disease (PD) is pervasive, with life‐impacting consequences. Yet, little is known about how functional connections between the basal ganglia and cortex relate to PD speech impairment (PDSI). Whole‐brain resting‐state connectivity analyses of basal ganglia nuclei can expand the understanding of PDSI pathophysiology.

**Methods:**

Resting‐state data from 89 right‐handed subjects were downloaded from the Parkinson's Progression Markers Initiative database. Subjects included 12 older healthy controls (“OHC”), 42 PD patients without speech impairment (“PDN”), and 35 PD subjects with speech impairment (“PDSI”). Subjects were assigned to PDN and PDSI groups based on the Movement Disorders Society Unified Parkinson's Disease Rating Scale (MDS‐UPDRS) Part III speech item scores (“0” vs. “1–4”). Whole‐brain functional connectivity was calculated for four basal ganglia seeds in each hemisphere: putamen, caudate, external globus pallidus (GPe), and internal globus pallidus (GPi). For each seed region, group‐averaged connectivity maps were compared among OHC, PDN, and PDSI groups using a multivariate ANCOVA controlling for the effects of age and sex. Subsequent planned pairwise *t*‐tests were performed to determine differences between the three groups using a voxel‐wise threshold of *p* < 0.001 and cluster‐extent threshold of 272 mm^3^ (FWE<0.05).

**Results:**

In comparison with OHCs, both PDN and PDSI groups demonstrated significant differences in cortical connectivity with bilateral putamen, bilateral GPe, and right caudate. Compared to the PDN group, the PDSI subjects demonstrated significant differences in cortical connectivity with left putamen and left GPi. PDSI subjects had lower connectivity between the left putamen and left superior temporal gyrus compared to PDN. In addition, PDSI subjects had greater connectivity between left GPi and three cortical regions: left dorsal premotor/laryngeal motor cortex, left angular gyrus, and right angular gyrus.

**Conclusions:**

The present findings suggest that speech impairment in PD is associated with altered cortical connectivity with left putamen and left GPi.

## 1 INTRODUCTION

Parkinson's disease (PD) is a progressive neurodegenerative disease involving degeneration of nigrostriatal dopaminergic pathways in the basal ganglia. While the impact of the disease on daily living typically manifests as impaired mobility, speech impairment is very common and can impair an individual's ability to communicate in daily life. It is estimated that 80%–90% of individuals with PD develop dysarthria over the course of the disease (Sapir, 2014), with deviant perceptual characteristics including monopitch, monoloudness, reduced stress, variable rate, short rushes of speech, and imprecise consonants (Duffy, 2013). As a result, the perceived intelligibility and naturalness of speech in individuals with PD can be negatively affected (Darley, Aronson, & Brown, 1969), leading to social withdrawal and impaired work‐related performance (Miller, Noble, Jones, & Burn, 2006).

In order to understand the neurobiology of speech impairments in PD, it is important to determine whether there are specific functional connections between the basal ganglia and cortex that uniquely contribute to speech symptoms. Cortico‐basal ganglia loops are critical for normal speech production. However, the specific contributions of basal ganglia circuits to speech production are not fully understood. Studies utilizing functional neuroimaging tools such as positron emission tomography (PET) and functional magnetic resonance imaging (fMRI) provide insight into the role of the basal ganglia in both normal and disordered speech. Of the subcortical nuclei comprising the basal ganglia pathways, the putamen is most commonly associated with speech and voice production in neuroimaging studies (Bohland & Guenther, 2006; Brown et al., 2009; Manes et al., 2014; Tourville & Guenther, 2011). Researchers have reported increased bilateral putamen activation during both speech and nonspeech vocal tract movements using fMRI (Brown et al., 2009; Chang, Kenney, Loucks, Poletto, & Ludlow, 2009; Parkinson et al., 2012). A recent PET study using D2/D3 receptor radioligands also demonstrated that speech production is accompanied by a left‐lateralized increase in endogenous dopamine release within the striatum (Simonyan, Herscovitch, & Horwitz, 2013), suggesting that left‐hemisphere striatal regions may in fact play a more important role than those in the right hemisphere. In addition to the striatum, the role of the pallidum has also been described in relation to normal speech production. A meta‐analysis of internal globus pallidus (GPi) and subthalamic nucleus coactivation maps revealed that the connectivity profiles of these two structures showed significant spatial overlap with brain regions involved in speech production, including the left putamen, left insula, and left ventrolateral nucleus of the thalamus (Manes et al., 2014). Both the globus pallidus and putamen have been incorporated in the Directions Into Velocity of Articulators (DIVA) computational model of speech production (Tourville & Guenther, 2011). Within this model, the globus pallidus and the putamen are involved in the initiation of speech movements through reciprocal functional connections with the supplementary motor area (SMA). Given the integral role of the basal ganglia in normal speech production, it is not surprising that basal ganglia disorders, such as PD and Huntington's disease, result in marked impairments in speech function. However, questions remain as to which functional connections between basal ganglia and cortex contribute to speech impairments in the presence of basal ganglia pathology and whether or not these are distinguishable from pathways contributing to nonspeech motor symptoms.

There are several cortical regions supporting normal speech production that could be affected by functional changes in the basal ganglia. It is well established that speech production involves the sensorimotor cortex, SMA, inferior frontal gyrus/ventral premotor cortex (PMv), superior temporal gyrus (STG)/Heschl's gyrus, and cerebellum (Brown, Ingham, Ingham, Laird, & Fox, 2005; Brown et al., 2009; Manes et al., 2014; Tourville & Guenther, 2011) These regions of the cortex are reliably active during speech and voice production tasks (Brown et al., 2005; Manes et al., 2014; Spaniol et al., 2009). Studies of whole‐brain resting‐state connectivity in PD have documented that the basal ganglia have abnormal connectivity to the cerebellum (Hacker, Perlmutter, Criswell, Ances, & Snyder, 2012) and motor cortices, including sensorimotor cortex (Baudrexel et al., 2011; Hacker et al., 2012; Kurani et al., 2015; Kwak et al., 2010), premotor cortex (Baudrexel et al., 2011), and SMA (Baudrexel et al., 2011; Hacker et al., 2012; Kwak et al., 2010). Given the critical role of these structures in speech production, it seems likely that changes in these connections contribute to speech problems in PD. However, it is also possible that speech impairments in PD involve abnormal basal ganglia connectivity to cortical brain regions that are not directly related to motor output, such as STG. Indeed, a study by Simonyan et al. (2013) found that the BOLD signal from the left anterior putamen was highly correlated with that of left STG when healthy individuals performed a sentence production task. In the presence of basal ganglia pathology, it is possible that in addition to cortical regions involved in motor control, changes in the functional connectivity of the basal ganglia structures to STG may also be involved with speech impairment in PD.

Functional connectivity analysis of resting‐state fMRI data provides a means for estimating the strength of functional basal ganglia connections to cortical and subcortical structures. By analyzing fMRI data in a task‐free context, researchers can make inferences about the intrinsic organization of functional brain networks that might otherwise be masked by the effects of task performance (Biswal, Yetkin, Haughton, & Hyde, 1995; Di Martino et al., 2008; Smith et al., 2009). While several studies have identified abnormal resting‐state basal ganglia connections in PD (Baudrexel et al., 2011; Hacker et al., 2012; Helmich et al., 2010; Kurani et al., 2015; Kwak et al., 2010; Wu et al., 2009), little work has been done to assess the relationship of these connections with speech symptoms. Two studies have used seed‐based resting‐state analysis to study the mechanisms of speech impairment in PD by comparing the connectivity of functionally relevant brain regions between PD and controls. New et al. (2015) found that PD subjects had reduced connectivity between right and left putamen after performing a seed to seed resting‐state connectivity analysis on thirteen regions involved in vocal motor control (Brown et al., 2005). The study further found that UPDRS Part III speech impairment scores were inversely correlated with right putamen connectivity to right cerebellum and left STG. A more recent study measured the whole‐brain resting‐state connectivity of three right hemisphere structures involved in emotional prosody (orofacial sensorimotor cortex, anterior cingulate cortex, and the caudate) and found that PD patients had reduced connectivity between the right caudate and right dorsolateral prefrontal cortex compared to healthy controls (Elfmarkova et al., 2016). Together these, two studies provide evidence for a link between striatal functional connectivity and impaired voice and prosodic function in PD. However, it is important to note that neither study limited its PD group to only those patients who presented with speech impairment. Further, it remains unclear whether speech impairment in PD may involve connectivity changes in other basal ganglia or cortical structures.

The current study was designed to extend previously published literature in two ways. First, as prior resting‐state studies of PD speech have only included striatal regions of the basal ganglia, we sought to investigate whether functional connections with the globus pallidus might also be linked to speech impairment in PD. Second, we chose to compare whole‐brain basal ganglia connectivity between three groups: older healthy control subjects (“OHC”), PD subjects with no speech impairment (“PDN”), and PD subjects with speech impairment (“PDSI”). By separating our PD subjects into PDN and PDSI groups, we sought to identify changes in functional basal ganglia connections that were specific to PD speech impairments and independent of more global, disease‐related motor impairments. If abnormal basal ganglia connectivity to motor cortices (sensorimotor cortex, SMA, premotor cortex) is in fact related to broader disease‐related changes in motor function, we would expect to see these connections emerge from the comparison of OHC and PDSI, but not in the comparison of PDN and PDSI. By contrast, we would expect to see group differences in basal ganglia connectivity with STG when comparing PDN to PDSI, as such a connection would presumably be independent of global motor severity. We thus predicted that striatal connectivity to motor cortices and STG would differ between PDSI and OHC groups, but that we would observe differences only in striatal–STG connectivity when comparing PDN to PDSI. Given that striatal dopamine release appears to be left‐lateralized during speech production (Simonyan et al. 2013), we further predicted that striatal connectivity differences between PDN and PDSI groups would occur in the left striatum. Although several speech models include the globus pallidus, none are predictive of whether there are resting‐state connectivity changes related to speech impairment in PD. As such, we made no specific predictions about connectivity between the globus pallidus and cortex.

## 2 MATERIALS AND METHODS

### 2.1 Data source

This study leverage a large sample of resting‐state fMRI data from the Parkinson's Progression Markers Initiative (PPMI; http://www.ppmi-info.org/; http://scicrunch.org/resolver/SCR_006431) in order to examine whether connections between the cortex and basal ganglia relate to speech impairment in PD. PPMI is an ongoing multi‐center project aimed at identifying biomarkers of PD through the longitudinal tracking of standardized clinical, imaging, and biometric assessments across 21 sites (16 US and five European sites) (Parkinson Progression Markers Initiative, 2011). PPMI follows the progression of 423 PD subjects who were newly diagnosed (<6 months) and not on antiparkinsonian medication at enrollment, as well as 196 age‐ and sex‐matched OHC subjects. While structural MRI data were collected for all PPMI subjects, the collection of resting‐state fMRI data was implemented at a later date across six of the twenty‐one sites resulting in fewer subjects with available resting‐state scans. For the purposes of this study, we searched the PPMI database for all subjects in the PD or OHC Cohorts who had received a resting‐state fMRI scan. At the time of analysis, we identified 90 PD and 21 OHC subjects who had participated in resting‐state fMRI scanning in addition to PPMI's standard data collection protocols. OHC subjects had completed their resting‐state scans at either their Baseline, Year 1, or Year 4 visit. PD subjects had completed their resting‐state scans at either their Baseline, Year 1, Year 2, or their visit prior to initiation of antiparkinsonian medication. From this sample of de‐identified subjects, we accessed resting‐state fMRI and structural MRI scans as well as clinical assessments describing PD features and severity, handedness, medication, cognitive function, and depression.

### 2.2 Inclusion/exclusion criteria

We restricted the final sample to include only right‐handed subjects whose fMRI scans passed our quality assurance review. We selected only right‐handed subjects to control for possible differences in the lateralization of speech and language representation in the cortex. For quality assurance, each resting‐state scan was required to have at least 90% of time points (188/208 volumes) with <0.5 mm frame wise displacement and with no outliers exceeding >5% root‐mean‐squared change in BOLD signal. Of the initial 111 subjects identified from the PPMI database, 13 were excluded because they were not right‐hand dominant and nine were excluded because their scans did not meet our quality assurance criteria.

### 2.3 PD group assignment

Speech impairment scores on the Movement Disorders Society Unified Parkinson's Disease Rating Scale (MDS‐UPDRS) Part III were used to assign PD subjects to either the PDN or PDSI group. While the scale provides only a coarse, global impression of speech severity, the availability of speech impairment scores through PPMI allows us to compare the resting‐state connectivity of PDN and PDSI groups using large sample sizes that are less feasible to collect in a prospective study. Under this item, speech impairment was rated on a scale of “0–4” (0 = “No speech problems”, 4 = “Most speech is difficult to understand or unintelligible”). PD subjects with a rating of “0” were assigned to the “PDN” group (*n* = 42) and PD subjects with a rating of “1–4” were assigned to the “PDSI” group (*n* = 35). Within the PDSI group, the median speech impairment score was “1” (33 subjects had a speech impairment rating of “1” and 2 subjects had a speech impairment rating of “2”, mean = 1.06).

### 2.4 Characteristics of participants

Resting‐state data were analyzed for 12 OHC, 42 PDN, and 35 PDSI subjects. Groups were similar across baseline characteristics. Table 1 demonstrates no differences in age, years of education, Geriatric Depression Scale (GDS), and Montreal Cognitive Assessment (MoCA) characteristics between the three groups; however, a less male gender preponderance was found for both PD groups compared to OHC. The identifiers for all analyzed subjects are presented in Table 2.

**Table 1:** PPMI subject characteristics for OHC, PDN and PDSI groups

Variable	OHC (*N* = 12)	PDN (*N* = 42)	PDSI (*N* = 35)	*p*‐value (PDN vs. PDSI)	*p*‐value (OHC vs. PDN)	*p*‐value (OHC vs. PDSI)
Age						
Mean (Min, Max)	65.33 (48, 83)	60.12 (39, 79)	64.14 (38, 77)	0.09	0.09	0.7
Gender						
Male	12 (100.0%)	28 (66.7%)	25 (71.4%)	0.65	**0.02** *	**0.04** *
Female	0 (0.0%)	14 (33.3%)	10 (28.6%)			
Education						
<13 years	0 (0.0%)	8 (19.0%)	8 (22.9%)	0.68	0.1	0.07
13–23 years	12 (100.0%)	34 (81.0%)	27 (77.1%)			
>23 years	0 (0.0%)	0 (0.0%)	0 (0.0%)			
MoCA^a^						
Mean (Min, Max)	27.83 (26, 30)	27.38 (18, 30)	26.85 (21, 30)	0.58	0.41	0.19
GDS						
Mean (Min, Max)	1.67 (0, 14)	2.26 (0, 10)	2.12 (0, 9)	0.79	0.63	0.71

*Notes*

GDS: geriatric depression scale; MoCA: Montreal cognitive assessment.

^a^Adjusted for years of education.

*p < 0.05, Chi‐squared test for independence.

Bold values indicate a statistically significant difference between the two groups.

**Table 2:** PPMI subject identifiers and scan visits for all analyzed OHC, PDN, and PDSI subjects

PPMI subject no.	Group	Visit (year)	PPMI subject no.	Group	Visit (year)	PPMI subject no.	Group	Visit (year)
3390	OHC	BL (baseline)	3378	PDN	V04 (year 1)	3119	PDSI	V04 (year 1)
4032	OHC	BL (baseline)	3380	PDN	V04 (year 1)	3123	PDSI	ST (year 1)
3310	OHC	V04 (year 1)	3758	PDN	V04 (year 1)	3327	PDSI	V04 (year 1)
3318	OHC	V04 (year 1)	3819	PDN	V04 (year 1)	3374	PDSI	V04 (year 1)
3769	OHC	V04 (year 1)	3825	PDN	V04 (year 1)	3575	PDSI	V04 (year 1)
3779	OHC	V04 (year 1)	3826	PDN	V04 (year 1)	3760	PDSI	V04 (year 1)
4018	OHC	V04 (year 1)	3828	PDN	V04 (year 1)	3771	PDSI	V04 (year 1)
3350	OHC	U01 (year 4)	3829	PDN	V04 (year 1)	3787	PDSI	V04 (year 1)
3351	OHC	U01 (year 4)	3832	PDN	V04 (year 1)	3822	PDSI	V04 (year 1)
3563	OHC	V10 (year 4)	3838	PDN	V04 (year 1)	3823	PDSI	V04 (year 1)
3369	OHC	U01 (year 4)	3863	PDN	V04 (year 1)	3830	PDSI	V04 (year 1)
3565	OHC	V10 (year 4)	4019	PDN	V04 (year 1)	3831	PDSI	V04 (year 1)
3130	PDN	BL (baseline)	4022	PDN	V04 (year 1)	3834	PDSI	V04 (year 1)
3134	PDN	BL (baseline)	3108	PDN	V06 (year 2)	3835	PDSI	V04 (year 1)
3383	PDN	BL (baseline)	3354	PDN	V06 (year 2)	4013	PDSI	V04 (year 1)
3385	PDN	BL (baseline)	3359	PDN	V06 (year 2)	3107	PDSI	V06 (year 2)
3392	PDN	BL (baseline)	3360	PDN	V06 (year 2)	3113	PDSI	V06 (year 2)
3593	PDN	BL (baseline)	3364	PDN	V06 (year 2)	3131	PDSI	V06 (year 2)
4030	PDN	BL (baseline)	3365	PDN	V06 (year 2)	3352	PDSI	V06 (year 2)
4035	PDN	BL (baseline)	3366	PDN	V06 (year 2)	3552	PDSI	V06 (year 2)
4038	PDN	BL (baseline)	3367	PDN	V06 (year 2)	3556	PDSI	V06 (year 2)
3118	PDN	V04 (year 1)	3585	PDN	V06 (year 2)	3574	PDSI	V06 (year 2)
3120	PDN	V04 (year 1)	3802	PDN	V06 (year 2)	3586	PDSI	V06 (year 2)
3122	PDN	V04 (year 1)	4021	PDN	V06 (year 2)	3587	PDSI	V06 (year 2)
3126	PDN	ST (year 1)	3332	PDSI	BL (baseline)	3800	PDSI	V06 (year 2)
3128	PDN	ST (year 1)	3386	PDSI	BL (baseline)	3808	PDSI	V06 (year 2)
3132	PDN	V04 (year 1)	3387	PDSI	BL (baseline)	3814	PDSI	V06 (year 2)
3371	PDN	V04 (year 1)	3589	PDSI	BL (baseline)	3818	PDSI	V06 (year 2)
3373	PDN	V04 (year 1)	3869	PDSI	BL (baseline)	4005	PDSI	V06 (year 2)
3375	PDN	V04 (year 1)	4034	PDSI	BL (baseline)			

### 2.5 PD characteristics

All PD subjects had a diagnosis of idiopathic PD and had clear evidence of a lateralized dopaminergic deficit on DaTSCAN^TM^. Subjects with a diagnosis of atypical Parkinsonism or those who showed no evidence of dopaminergic deficit were not included in the data analysis. The PDN and PDSI groups had similar baseline PD characteristics, including family history, Hoehn & Yahr scale, PD subtype, MDS‐UPDRS scores, and levodopa equivalent daily dose (LEDD; Tomlinson et al., 2010); however, those in the PDSI group were more likely to present with right‐lateralized motor symptoms. Disease characteristics of the PDN and PDSI groups are provided in Table 3. While all PD Cohort subjects were de novo when they enrolled in the PPMI study, some subjects were on dopaminergic therapy at the time of their resting‐state fMRI scans. This resulted in a mixed group of subjects relative to PD medication use. Unlike limb motor symptoms, the effect of dopamine treatment on voice or speech is neither robust nor consistent (Pinto et al., 2004; Schulz & Grant, 2000). We thus chose to include both medicated and nonmedicated PD subjects. We performed additional analyses to look for potential relationships between LEDD and basal ganglia connectivity should they exist. PD subjects who had begun taking antiparkinsonian medication were scanned while on medication, per PPMI protocol. For those subjects, we used on‐medication MDS‐UPDRS Part III scores to determine group assignment and motor severity. Patients were typically scanned on the same day as their motor evaluation; however, this varied based on scheduling considerations and scanner availability.

**Table 3:** Parkinson's disease characteristics for PDN and PDSI groups

Variable	PDN (*N* = 42)	PDSI (*N* = 35)	*p*‐value (PDN vs. PDSI)
Family history of PD			
Family members w/PD	14 (33.3%)	11 (22.9%)	0.31
No family members w/PD	28 (66.7%)	27 (77.1%)	
MDS‐UPDRS			
MDS‐UPDRS total score	30.24	35.55	0.11
MDS‐UPDRS part I	6.4	6.77	0.75
MDS‐UPDRS part II	6.62	7.37	0.47
MDS‐UPDRS part III (motor exam)	17.21	21.17	0.06
Hoehn & Yahr			
Stage 0	0 (0.0%)	0 (0.0%)	0.54
Stage 1	13 (31.0%)	11 (31.4%)	
Stage 2	29 (69.0%)	23 (65.7%)	
Stage 3–5	0 (0.0%)	1 (2.9%)	
TD/PIGD classification			
TD	31 (73.8%)	19 (54.3%)	0.14
PIGD	5 (11.9%)	10 (28.6%)	
Indeterminate	6 (14.3%)	6 (17.1%)	
Side most affected			
Left	20 (47.6%)	9 (25.7%)	**0.00** a
Right	20 (47.6%)	26 (74.3%)	
Symmetric	2 (4.8%)	0 (0.0%)	
PD medication usage			
PD medication	26 (61.9%)	24 (68.6%)	0.54
No PD medication	16 (38.1%)	11 (31.4%)	
Levodopa equivalent daily dose			
Mean (Min, Max)	219.56 (0, 600)	231.65 (0, 760)	0.81

a *p* < 0.05, Chi‐squared test for independence. Bold values indicate a statistically significant difference between the two groups.

### 2.6 Image acquisition

Structural and functional brain images in the PPMI dataset were acquired using 3T Siemens TIM Trio MRI scanners across six sites with standardized imaging protocols. T1‐weighted 3D anatomical scans were acquired in the sagittal plane using a MPRAGE GRAPPA protocol (TR = 2,300 ms, TE = 2.98 ms, flip angle = 9°, slice thickness = 1 mm, FOV = 256 mm × 256 mm, voxel size = 1 mm isotropic). BOLD T2*‐weighted echo‐planar images were acquired in 40 ascending slices (TR = 2,400 ms, TE = 25 ms, flip angle = 80°, slice thickness = 3.3 mm, no gap between slices, FOV = 222 mm × 222 mm, voxel size = 3.29 mm × 3.29 mm × 3.3 mm). Each resting‐state scan collected 212 volumes (8 min, 29 s). During all resting‐state scans, subjects were asked to relax, keep their eyes open, and to keep their mind free of thought (Van Dijk et al., 2010).

### 2.7 Preprocessing

All data were preprocessed with a custom pipeline using AFNI and SPM 12 tools. The first four resting‐state volumes were discarded to allow the MRI signal to reach equilibrium, leaving a total of 208 time points. Next, resting‐state functional MRI scans were despiked, corrected for slice timing, and realigned to the reference volume (first time point) in AFNI. Time points with excessive motion (>0.5 mm) and outliers (>5% root‐mean‐squared change in the BOLD signal) were then identified for censoring at a later stage. T1‐weighted structural MRI scans were coregistered to resting‐state functional scans in AFNI before being segmented into white matter, gray matter, and cerebral spinal fluid (CSF) tissue classes in SPM12. As the default tissue probability priors in SPM12 often misclassify basal ganglia nuclei as white matter (particularly the globus pallidus), we took additional steps to subtract these nuclei from the white matter mask. Using the @Anaticor tool in AFNI, we then regressed out nuisance white matter and CSF signals as well as motion and motion derivatives. Resting‐state scans underwent additional linear detrending and band‐pass filtering (0.01–0.1 Hz). The data were then censored to remove time points that had >0.5 mm frame wise displacement or >5% root‐mean‐squared change in the BOLD signal. The functional data were then smoothed to reach a full‐width‐half maximum of 6 mm using 3dBlurtoFWHM in AFNI.

### 2.8 Basal ganglia seed definitions

**Figure 1:**
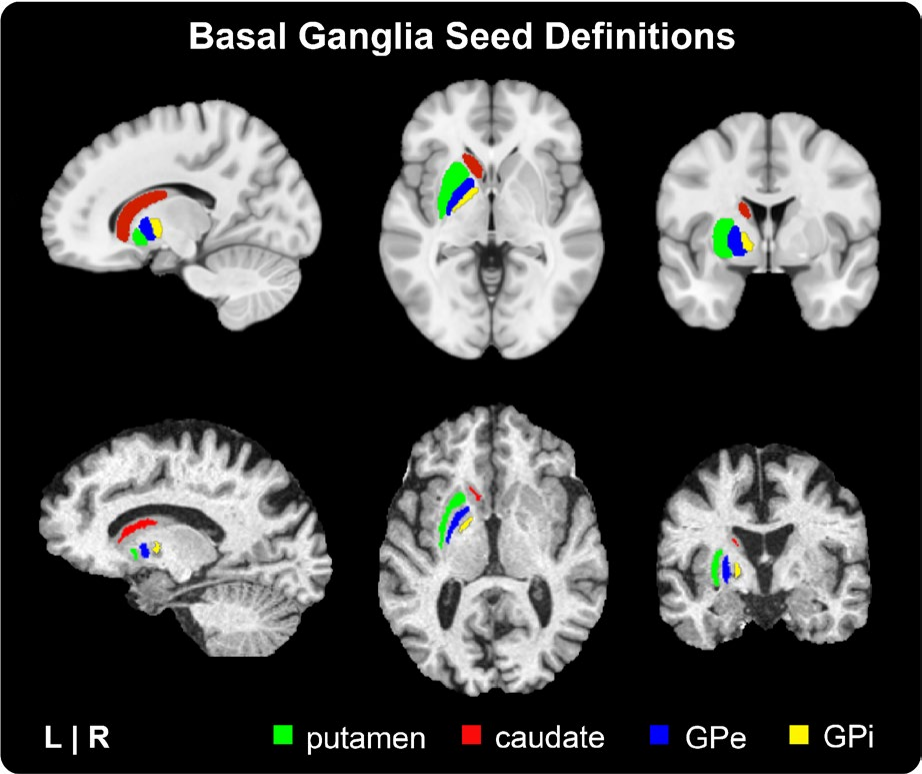
Basal ganglia seed definitions. Masks for the putamen, caudate, GPe, and GPi seeds were derived from the Basal Ganglia Human Area Template (BGHAT). Top row: BGHAT template regions overlaid onto the MNI template brain. Bottom row: BGHAT regions warped, eroded, and overlaid onto an individual subject's T1‐weighted structural MRI. Abbreviations: external globus pallidus (GPe), internal globus pallidus (GPi)

We analyzed the functional connectivity of four basal ganglia seeds in each hemisphere. These were bilateral caudate, putamen, GPe, and GPi. The Basal Ganglia Human Area Template (BGHAT) was used to define the boundaries of seed locations for the caudate, putamen, GPe, and GPi in MNI space (Prodoehl, Yu, Little, Abraham, & Vaillancourt, 2008). Once defined in MNI space, basal ganglia seed definitions were transformed into subject‐space to define individualized seed regions for each subject. To do this, a single nonlinear transform (comprised of affine and nonlinear warps) was calculated in order to normalize the coregistered T1‐weighted structural scan to the MNI 2009c symmetric template brain (Fonov, Evans, McKinstry, Almli, & Collins, 2009). The inverse transform was then applied to basal ganglia seed definitions, transforming them from MNI to subject‐space (Figure 1). The outer edge of each seed region was then eroded by 1 mm to minimize partial volume signal from neighboring white matter and CSF. To confirm placement, we performed visual inspection of putamen, caudate, GPe, and GPi seeds on each subject's T1‐weighted structural scan (coregistered with functional resting‐state data).

### 2.9 Functional connectivity

Connectivity maps for each seed were calculated within each subject. Pearson correlations were calculated in subject‐space to describe the connectivity between the seed and each voxel within the whole‐brain mask. The resulting Pearson's *r* correlation maps were then converted to a *Z*‐score map via Fisher's transform. Each *Z*‐score map was then warped to 2 mm × 2 mm × 2 mm MNI space in preparation for a group analysis. Group‐averaged *Z*‐score maps were calculated for OHC, PDN, and PDSI groups for statistical comparisons.

### 2.10 Statistical analysis

For each of the eight basal ganglia seeds, we performed a three‐group analysis of covariance (ANCOVA) controlling for the effects of age and sex using the 3dMVM tool in AFNI. Planned, pairwise *t*‐tests were then performed between‐groups (OHC vs. PDN, OHC vs. PDSI, PDN vs. PDSI). To determine cluster‐wise statistical thresholds for our between‐groups comparisons, we first estimated the smoothness of noise in the resting‐state scans using the spatial autocorrelation function in AFNI (Cox, Reynolds, & Taylor, 2016). This new approach to smoothness estimation was developed to address the recently identified issue of inflated false‐positive rates resulting from the inappropriate smoothing calculations used by several imaging software packages (Cox et al., 2016; Eklund, Nichols, & Knutsson, 2016). Data smoothness was calculated for each subject individually using the preprocessed resting‐state scans prior to nuisance signal regression in Anaticor (i.e., before removing noise) with the warp to MNI space applied. Smoothness estimates were then averaged across all subjects and entered into AFNI's 3dClustSim program. To reach a family‐wise error (FWE) level of 0.05, statistical significance was defined using a voxel‐wise threshold of *p *<* *0.001 and a cluster‐wise threshold of 34 voxels (34 voxels × 2 mm^3 ^= 272 mm^3^).

To control for differences in brain coverage across subjects (specifically, clipping of slices at the top and bottom of the brain), our results were restricted to a group mask limited to voxels with at least 90% coverage across all subjects.

As motor severity and LEDD were not appropriate covariates to include with the OHC group, we conducted a two‐group analysis using only the PD subjects to determine whether the motor severity or LEDD might influence the results of the PDN vs. PDSI comparisons within the three‐group ANCOVA. The second set of ANCOVAs was performed on PDN and PDSI groups while controlling for the effects of age, sex, LEDD, and motor severity (MDS‐UPDRS Part III) using the same statistical threshold. The results did not differ from PDN versus PDSI comparisons in the three‐group ANCOVA.

### 2.11 Correlation with motor severity and PD medication

We also performed correlational analyses for PDN and PDSI groups separately to examine the relationship between connectivity values and MDS‐UPDRS Part III aggregate scores as well as LEDD. Within each significant cluster, we extracted the averaged *Z*‐score for each individual PDN and PDSI subject. The subject‐level *Z*‐scores were correlated with MDS‐UPDRS Part III scores within the PDN and PDSI groups using a Pearson's *r* correlation (*p *<* *0.05). To examine whether there was a relationship between group connectivity differences and use of antiparkinsonian medication, we further correlated *Z*‐scores of medicated PDN and PDSI subjects with LEDD using a Pearson's *r* correlation (*p *<* *0.05).

## 3 RESULTS

Our results are organized by seed region (left putamen, right putamen, left caudate, right caudate, left GPe, right GPe, left GPi, right GPi).

### 3.1 Left putamen

**Figure 2:**
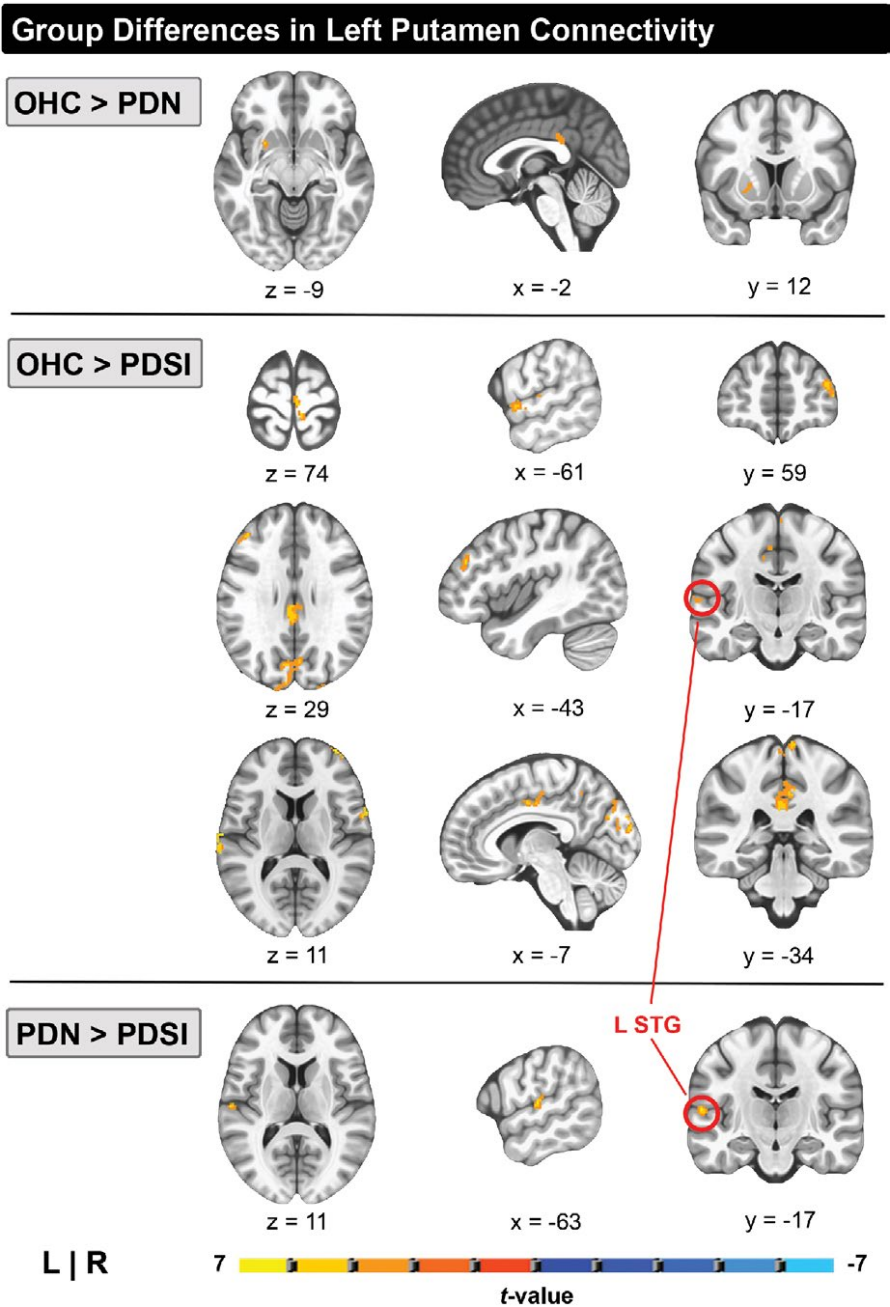
Pairwise group differences in whole‐brain resting‐state functional connectivity of the left putamen (*p *<* *0.001, cluster size >272 mm^3^ *,* FWE<0.05). Top row: Areas of reduced left putamen connectivity in PD versus HC. Middle row: Areas of reduced left putamen connectivity in PDSI versus HC. Bottom row: Areas of reduced left putamen connectivity in PDSI versus PD. The red circle indicates a region in the left posterior STG with reduced connectivity in PDSI compared to both HC and PD groups. Abbreviations: superior temporal gyrus (STG)

**Figure 3:**
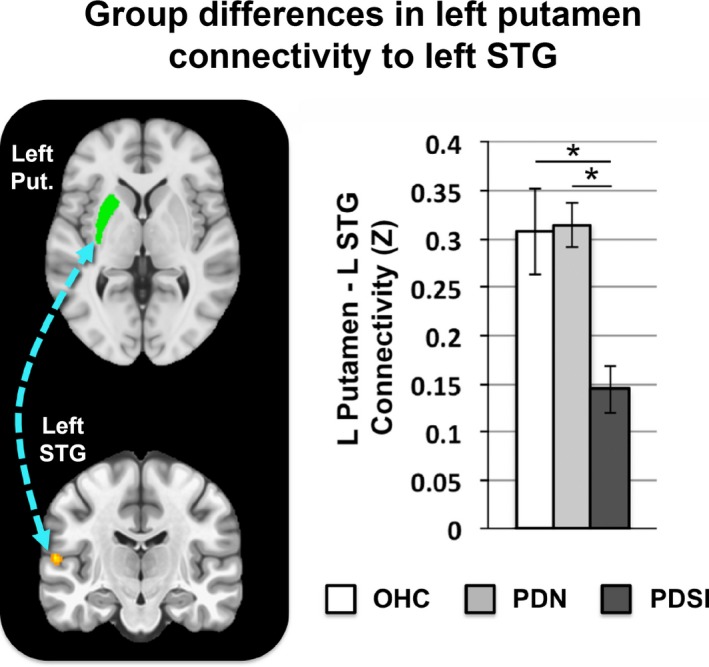
Mean functional connectivity between left putamen and left STG across OHC, PDN, and PDSI groups. The connectivity values for each group represent the mean *Z*‐score within a cluster‐derived mask of left STG (OHC:* Z *=* *0.307, PDN:* Z *=* *0.315, PDSI:* Z *=* *0.144). Significance was derived from our voxel‐wise analysis (*p < 0.001, cluster size >272 mm^3^, FWE<0.05). Abbreviations: putamen (Put.), superior temporal gyrus (STG)

A multivariate analysis of left putamen connectivity revealed a significant effect for group membership. Differences in left putamen connectivity were observed in all three pairwise comparisons (OHC vs. PDN, OHC vs. PDSI, PDN vs. PDSI) as shown in Table 4. Compared to the OHC group, PDN subjects had lower functional connectivity between the left putamen and left posterior cingulate cortex (Figure 2, top row, middle column). In addition, the PDN group had lower connectivity between the left putamen seed and a subset of voxels within the left putamen (Figure 2, top row, left and right columns). When compared to the OHC group, the PDSI group also had reduced connectivity between left putamen and left posterior cingulate cortex (Figure 2, fourth row, right column). Reductions in the connectivity between the left putamen seed and the subset of voxels within the left putamen were found when only the voxel‐wise threshold was applied; however, the cluster did not meet our cluster‐extent threshold when comparing OHC versus PDSI. In addition to the posterior cingulate cortex, the PDSI group had reduced connectivity between the left putamen and several other cortical regions, including sensorimotor cortex (Figure 2, second row, left column) cingulate motor area (Figure 2, fourth row, middle column), and two clusters in the left STG (Figure 2, middle row, right column). Compared to the PDN group, the PDSI group had significantly lower connectivity between left putamen and a single cluster in left STG (Figure 2, bottom row). Figure 3 summarizes the mean functional connectivity (*Z*) between the left putamen seed and left STG cluster, illustrating that there is no difference between OHC versus PDN subjects, but that connectivity is significantly lower in PDSI compared to both OHC and PDN.

**Table 4:** Pairwise group differences in basal ganglia connectivity

	Comparison	Brain region(s)	Size (mm^3^)	MNI coordinates (peak)			*t*‐value
				*x*	*y*	*z*	
Seed	Between‐group differences						
Left Putamen	OHC > PDN	L Putamen	288	−25	9	−9	4.229
		L Posterior Cingulate	272	−1	−35	27	4.118
	OHC > PDSI	L Posterior Cingulate	4,240	−1	−31	27	5.116
		R Cuneus	2,800	7	−71	27	4.727
		R Middle Frontal Gyrus	1,056	41	61	7	5.09
		R Paracentral Lobule	1,032	7	−33	77	4.617
		L Middle Cingulate Cortex	632	−7	−3	41	5.152
		L Superior Temporal Gyrus	592	−63	−7	−1	4.857
		L Middle Frontal Gyrus	464	−41	41	27	4.587
		R Precuneus	456	7	−59	53	8.586
		L Superior Temporal Gyrus	312	−69	−25	13	4.659
		R Superior Occipital Gyrus	304	25	−95	27	4.894
	PDN > PDSI	L Superior Temporal Gyrus	272	−59	−17	11	5.643
Right Putamen	OHC > PDN	R Middle Cingulate	824	1	−31	33	4.271
	OHC > PDSI	L Middle Cingulate	2440	1	−41	45	5.507
		L Superior Frontal Gyrus	984	−33	65	13	5.508
		R Paracentral Lobule	776	3	−25	75	5.373
		R Superior Frontal Gyrus	752	35	65	9	7.446
		L Cuneus	688	−5	−93	21	4.468
		R Lingual Gyrus	632	15	−41	−5	4.894
		L SMA	544	−11	−15	49	4.809
		R Superior Medial Gyrus	392	7	27	59	4.244
		R Cuneus	384	19	−83	43	4.036
		L Parahippocampal Gyrus	336	−27	−43	−9	4.588
		L Middle Temporal Gyrus	312	−69	−25	−5	4.825
	PDN > PDSI	‐	‐	‐	‐	‐	‐
Left Caudate	OHC > PDN	‐	‐	‐	‐	‐	‐
	OHC > PDSI	‐	‐	‐	‐	‐	‐
	PDN > PDSI	‐	‐	‐	‐	‐	‐
Right Caudate	OHC > PDN	L SMA	416	−7	15	61	4.871
	OHC > PDSI	L SMA	1,432	−9	15	55	5.592
		R Inferior Temporal Gyrus	392	57	−59	−11	4.931
	PDN > PDSI	‐	‐	‐	‐	‐	‐
Left GPe	OHC > PDN	R Cuneus	312	5	−77	35	4.469
	OHC > PDSI	L Middle Occipital Gyrus	2,376	−25	−85	17	5.261
		L SMA	472	−9	−15	49	5.412
	PDN > PDSI	‐	‐	‐	‐	‐	‐
Right GPe	OHC > PDN	R Precuneus	576	3	−77	37	4.965
	OHC > PDSI	R Paracentral Lobule	408	3	−37	71	5.194
		L SMA	392	−11	−15	51	5.403
		R Cuneus	272	1	−77	35	4.305
	PDN > PDSI	‐	‐	‐	‐	‐	‐
Left GPi	OHC > PD	‐	‐	‐	‐	‐	‐
	OHC > PDSI	‐	‐	‐	‐	‐	‐
	PDN > PDSI	L Angular Gyrus	488	−39	−63	41	−4.492
		L Precentral Gyrus (PMd/LMC)	464	−51	7	43	−4.573
		R Angular Gyrus	352	37	−63	41	−4.878
Right GPi	OHC > PDN	‐	‐	‐	‐	‐	‐
	OHC > PDSI	‐	‐	‐	‐	‐	‐
	PDN > PDSI	‐	‐	‐	‐	‐	‐

*Notes*

LMC: laryngeal motor cortex; PMd: dorsal premotor cortex; SMA: supplemental motor area; FWE = 0.05. *p* < 0.001, cluster extent >272 mm^3^ (34 voxels).

### 3.2 Right putamen

A significant group effect was identified for the right putamen. Differences in right putamen connectivity were observed in two of the three pairwise comparisons (OHC vs. PDN and OHC vs. PDSI). PDN subjects had lower connectivity between the right putamen and the right middle cingulate compared to OHC subjects. When compared to OHC subjects, the PDSI group demonstrated widespread reductions in right putamen connectivity to cortical regions, including left cingulate motor area, left SMA and right sensorimotor cortex. However, there were no statistically significant differences in right putamen connectivity between subjects in the PDN and PDSI groups (Table 4).

### 3.3 Left caudate

No significant group effects were found for the left caudate seed.

### 3.4 Right caudate

A significant group effect was found for the right caudate seed. Differences in right caudate connectivity were observed in two of the three pairwise comparisons (OHC vs. PDN and OHC vs. PDSI). Compared to the OHC group, PDN subjects had lower connectivity of the right caudate to left SMA. Differences were also observed between OHC and PDSI groups, with the PDSI subjects demonstrating reduced connectivity of the right caudate with left SMA and right inferior temporal gyrus. There were no statistically significant differences in right caudate connectivity between PDN and PDSI groups.

### 3.5 Left GPe

A significant group effect was found for the left GPe seed. Differences in left GPe connectivity were observed in two of the three pairwise comparisons (OHC vs. PDN and OHC vs. PDSI). Compared to the OHC group, PDN subjects had lower connectivity between the left GPe and the right cuneus. The PDSI group had lower left GPe connectivity to left middle occipital gyrus and left SMA compared to OHC subjects. No differences were found between PDN and PDSI groups.

### 3.6 Right GPe

A significant group effect was found for the right GPe seed. Differences in right GPe connectivity were observed in two of the three pairwise comparisons (OHC vs. PDN and OHC vs. PDSI). PDN subjects had lower connectivity between right GPe and right precuneus compared to OHC subjects. In addition, the PDSI group demonstrated lower right GPe connectivity to right paracentral lobule, left SMA, and left cuneus compared to the OHC group. No differences in right GPe connectivity were observed between PDN and PDSI groups.

### 3.7 Left GPi

**Figure 4:**
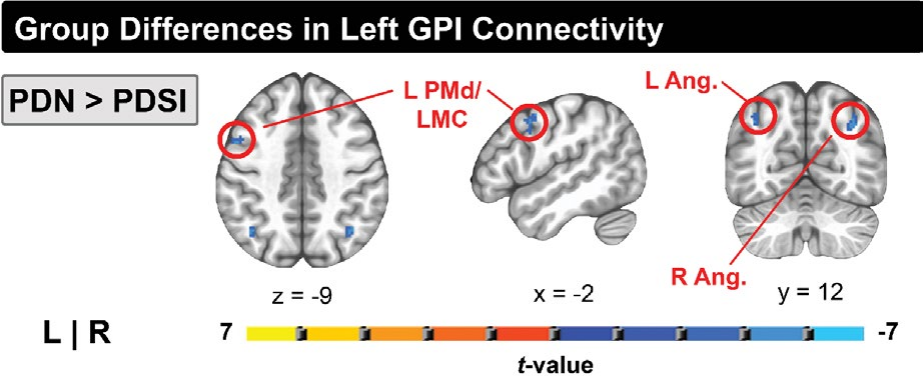
Pairwise group differences in whole‐brain resting‐state functional connectivity of the left GPi (*p* < 0.001, cluster size >272 mm^3^, FWE<0.05). Shown in blue are regions of increased functional connectivity of left GPi in PDSI versus PD. The red circle indicates a region on the precentral gyrus corresponding to the dorsal premotor cortex/laryngeal motor cortex. Abbreviations: dorsal premotor cortex (PMd), laryngeal motor cortex (LMC), internal globus pallidus (GPi)

**Figure 5:**
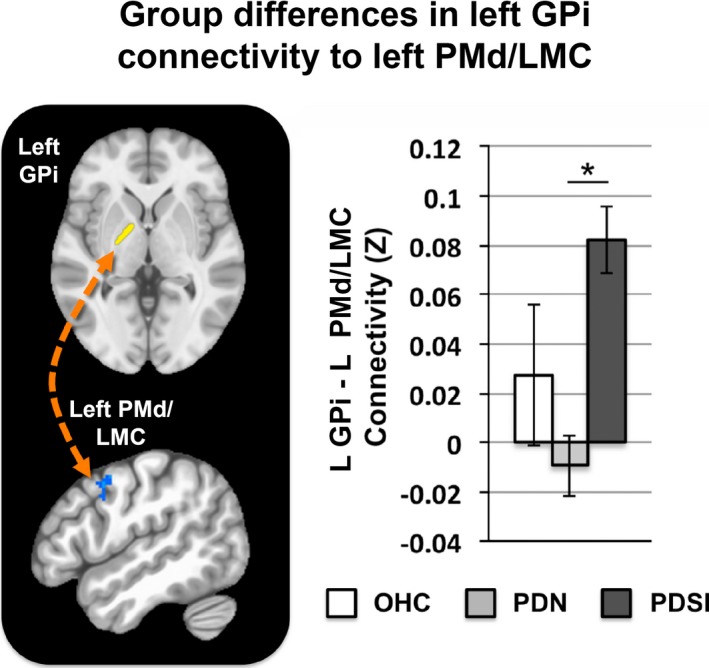
Mean functional connectivity between left GPi and left PMd across OHC, PDN, and PDSI groups. The connectivity values for each group represent the mean *Z*‐score within a cluster‐derived mask of left PMd/LMC (OHC:* Z *=* *0.028, PDN:* Z *=* *−0.009, PDSI:* Z *=* *0.082). Significance was derived from our voxel‐wise analysis (*p < 0.001, cluster size >272 mm^3^, FWE<0.05). Abbreviations: dorsal premotor cortex (PMd), laryngeal motor cortex (LMC)

**Figure 6:**
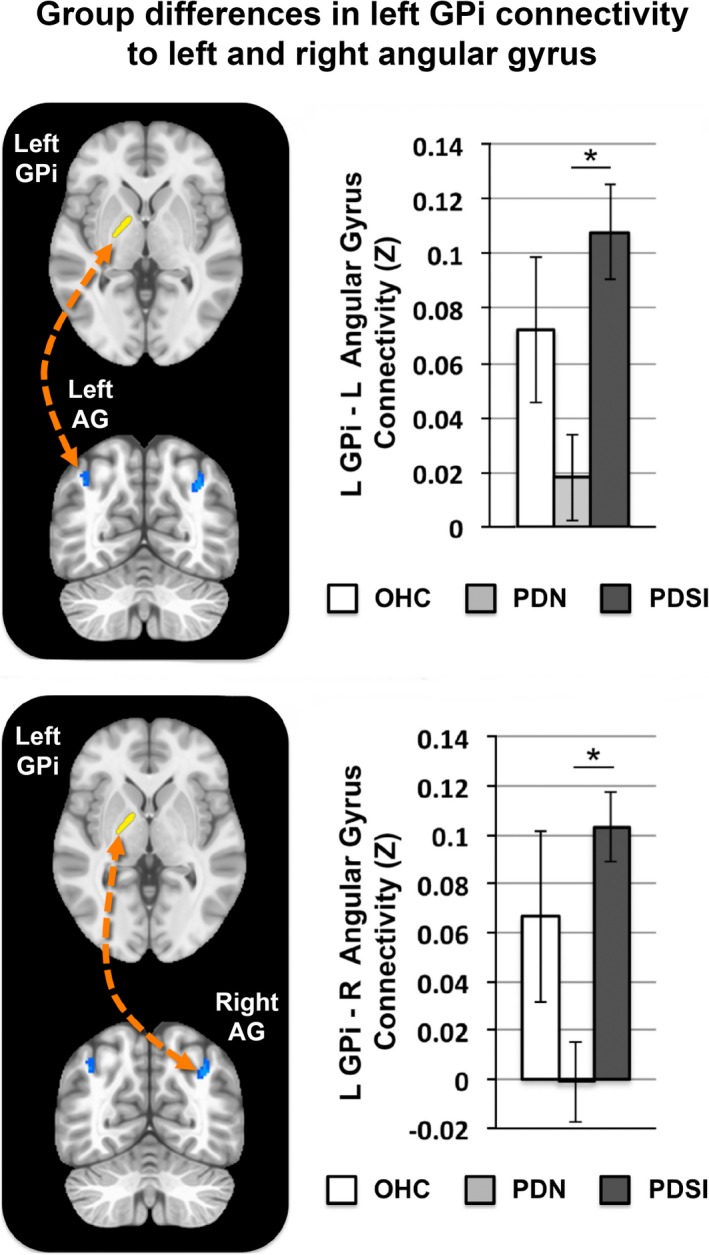
Top: Mean functional connectivity between left GPi and left angular gyrus (AG) across OHC, PDN, and PDSI groups. The connectivity values for each group represent the mean *Z*‐score within a cluster‐derived mask of left angular gyrus (OHC:* Z *=* *0.072, PDN:* Z *=* *0.018, PDSI:* Z *=* *0.108). Bottom: Mean functional connectivity between left GPi and right AG. The connectivity values for each group represent the mean *Z*‐score within a cluster‐derived mask of right angular gyrus (OHC:* Z *=* *0.067, PDN:* Z *=* *−0.001, PDSI:* Z *=* *0.103). Significance was derived from our voxel‐wise analysis (*p < 0.001, cluster size >272 mm^3^, FWE<0.05)

A significant group effect was found for the left GPi seed. Differences in left GPi connectivity were observed in one of the three pairwise comparisons (PDN vs. PDSI) as shown in Table 4. When compared to the PDN group, our analysis revealed that the PDSI group had stronger left GPi connectivity with a region of the left precentral gyrus, corresponding to left dorsal premotor cortex (PMd) and dorsolateral laryngeal motor cortex (LMC; Figure 4, left and middle columns) as well as stronger left GPi connectivity with left and right angular gyrus (Figure 4, right column). No significant differences were observed between the OHC and PDN group or between the OHC and PDSI group. Figures 5 and 6 summarize the mean functional connectivity (*Z*) of left GPi connectivity to left PMd/LMC (Figure 5) and bilateral angular gyrus (Figure 6), illustrating that connectivity is no different between OHC versus PDN subjects or OHC versus PDSI subjects, but that it is significantly higher in PDSI compared to PDN. It is important to point out that although statistically significant differences were not found for the OHC versus PDN and the OHC versus PDSI comparisons, Figures 5 and 6 (top and bottom panels) show that the mean *Z*‐score of the OHC subjects does look different when compared to PDN and PDSI. This raises the possibility that we did not have the sensitivity to detect a significant difference. For the two connections between left GPi and angular gyrus, a post hoc seed to seed analysis showed that the mean connectivity values approached significance for the comparison of OHC and PDN subjects (left GPi–left angular gyrus: *t *=* *1.742, *p *=* *0.098; left GPi–right angular gyrus: *t *=* *1.753, *p *=* *0.099). A post hoc sample size estimate demonstrated that we would need the following sample sizes to detect significant differences for these connections: 114 subjects per group (OHC vs. PDN) and 70 subjects per group (OHC vs. PDSI) for the left PMd/LMC connection; 70 subjects per group (OHC vs. PDN) and 109 subjects per group (OHC vs. PDSI) for the left angular gyrus connection; and 44 subjects per group (OHC vs. PDN) and 237 subjects per group (OHC vs. PDSI) for the right angular gyrus connection. We address this point in the discussion**.**

### 3.8 Right GPi

No significant group effects were found for the right GPi seed.

### 3.9 Correlation with motor severity and PD medication

**Figure 7:**
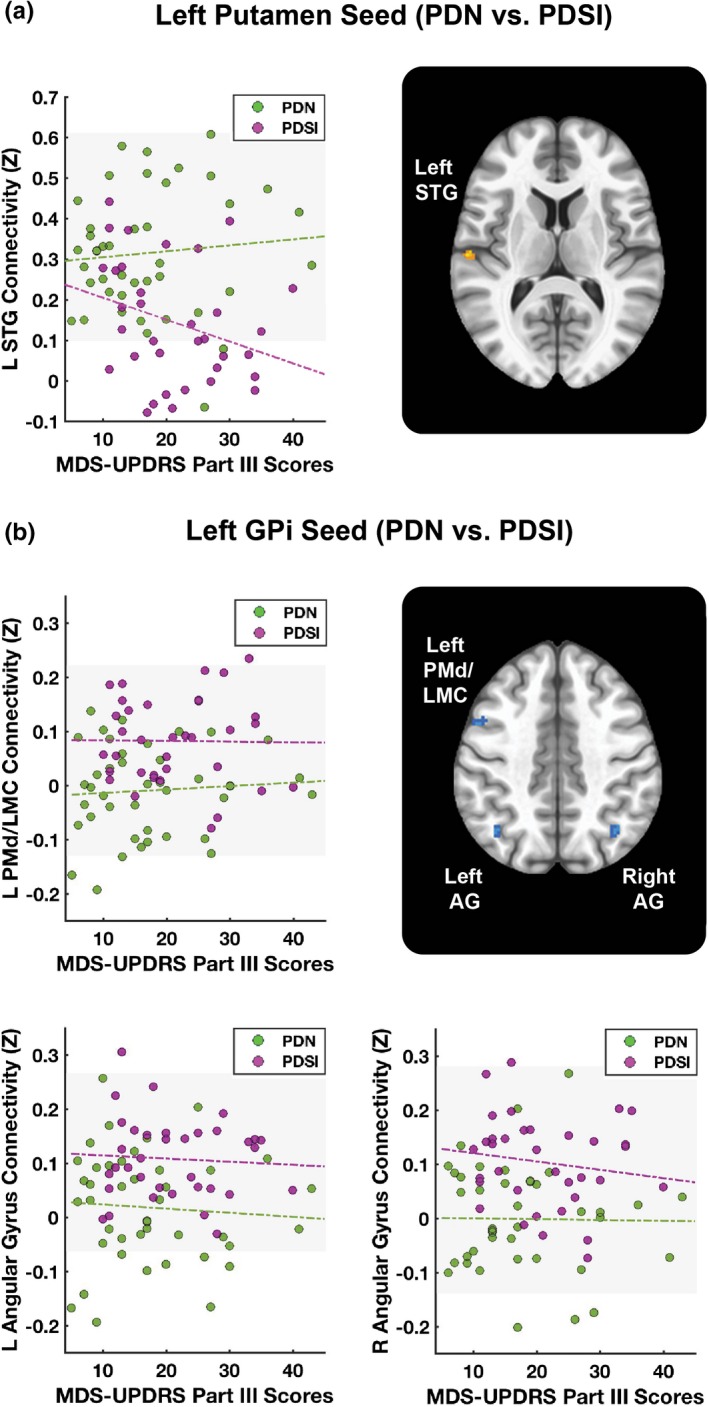
Functional connectivity and motor severity scores. Scatter plots depict functional connectivity scores (*Z*) plotted against motor severity scores (MDS‐UPDRS Part III). Green dashed lines represent a linear fit of the data for PDN subjects. Purple dashed lines represent a linear fit of the data for PDSI subjects. A) Motor severity correlations for left putamen–left STG connection. B) Motor severity correlations for left GPi–left PMd/LMC, left GPi–left angular gyrus, and left GPi–right angular gyrus connections. Abbreviations: superior temporal gyrus (STG), internal globus pallidus (GPi), dorsal premotor cortex (PMd), laryngeal motor cortex (LMC), Movement Disorders Society—Unified Parkinson's Disease Rating Scale (MDS‐UPDRS)

The comparison of PDN and PDSI subjects revealed group differences in four distinct functional connections: (a) left putamen–left STG, (b) left GPi–left PMd/LMC, (c) left GPi–left angular gyrus, (d) left GPi–right angular gyrus. To determine whether the strength of these connections was related to motor symptom severity, we first extracted the mean connectivity scores for each of these four seed‐cluster pairs, as described above. We then used a Pearson's *r* calculation to correlate mean connectivity values with MDS‐UPDRS Part III scores within PDN and PDSI groups, applying a statistical threshold of *p *<* *0.05. MDS‐UPDRS Part III motor scores did not correlate significantly with the connectivity of left putamen–left STG (PDN: *r *=* *0.093, *p *=* *0.560; PDSI: *r *=* *−0.3044, *p *=* *0.076), left GPi–left PMd /LMC (PDN: *r *=* *0.075, *p *=* *0.639; PDSI: *r *=* *0.012, p = 0.944), left GPi–left angular gyrus (PDN: *r *=* *−0.071, *p *=* *0.655; PDSI: *r *=* *−0.065, *p *=* *0.711), or left GPi–right angular gyrus (PDN: *r *=* *−0.014, *p *=* *0.930; PDSI: *r *=* *−0.1518, *p *=* *0.384). Figure 7 depicts the mean seed to cluster connectivity (*Z*) plotted against MDS‐UPDRS Part III scores for each or the four seed‐cluster pairs.

**Figure 8:**
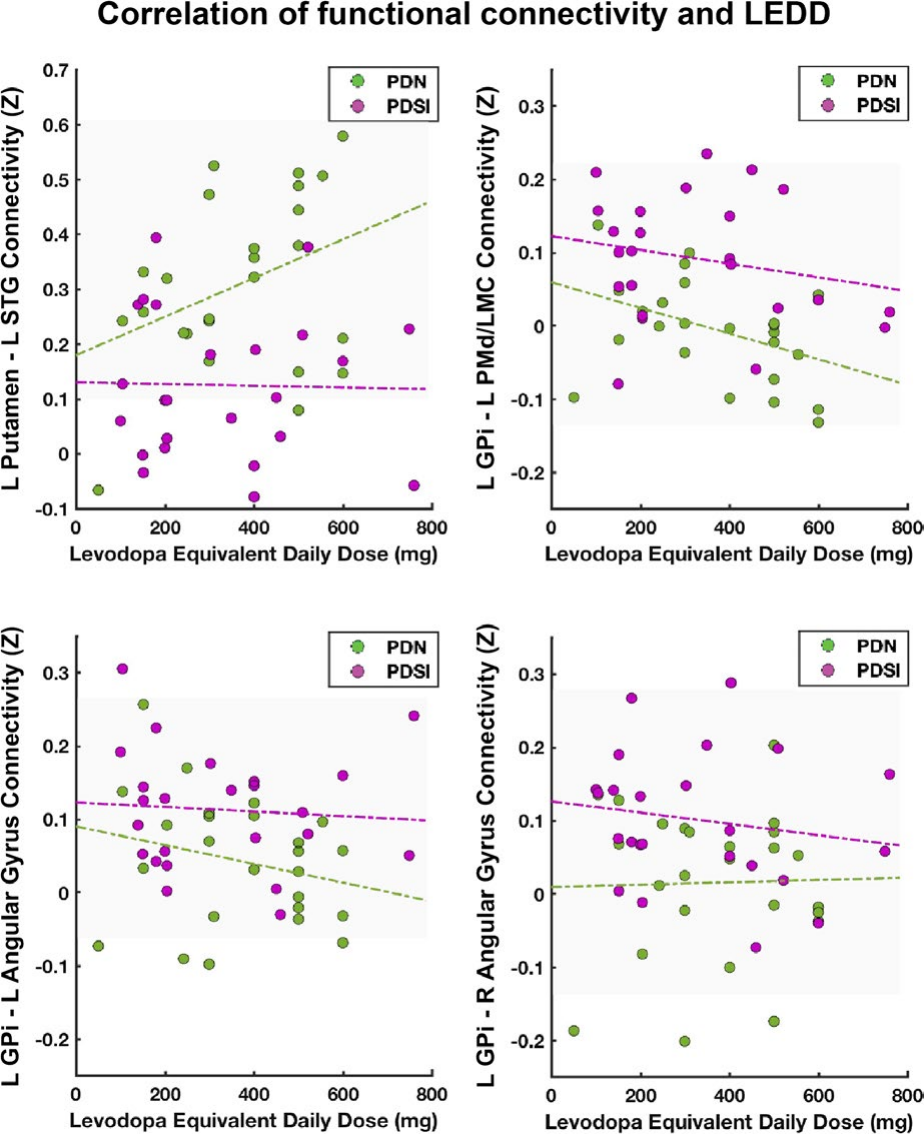
Functional connectivity and levodopa equivalent daily dose (LEDD). Scatter plots depict functional connectivity scores (*Z*) plotted against LEDD (mg). Green dashed lines represent a linear fit of the data for PDN subjects. Purple dashed lines represent a linear fit of the data for PDSI subjects

To determine whether group differences in connectivity strength were related to medication effects, we further correlated LEDD with the strength of the same four functional connections within medicated PDN and PDSI groups. This analysis revealed that the connectivity strength between left GPi and left PMd/LMC was inversely correlated with LEDD within the PDN group (*r *=* *−0.403, *p *=* *0.046*, Figure 8—top right corner), but not within the PDSI group (*r *=* *−0.213, *p *=* *0.317). LEDD did not correlate significantly with the connectivity of left putamen–left STG (PDN: *r *=* *0.367, *p *=* *0.072; PDSI: *r *=* *−0.024, *p *=* *0.911), left GPi–left angular gyrus (PDN: *r *=* *−0.240, *p *=* *0.249; PDSI: *r *=* *−0.076, *p *=* *0.724), or left GPi–right angular gyrus (PDN: *r *=* *0.025, *p *=* *0.905; PDSI: *r *=* *−0.166, *p *=* *0.439).

## 4 DISCUSSION

This study identified differences in functional basal ganglia connections between OHC, PDN, and PDSI subjects, which furthers our understanding of the neural processes contributing to speech production difficulties in PD. These differences can be summarized by five key findings. First, our seed to whole‐brain analyses identified a connection between left putamen and left STG that was significantly reduced in PDSI compared to both OHC and PDN groups (Figures 2 and 3). Second, our analyses identified three connections between left GPi and cortex in which PDSI subjects had increased connectivity compared to the PDN group (Figures 4, 5, 6). Third, the results of our PDN versus PDSI comparisons were not related to severity of motor impairments (Figure 7). Fourth, functional connectivity between left GPi and left PMd/LMC was inversely correlated with LEDD in the PDN, but not the PDSI group (Figure 8). Finally, we observed that group differences between PDN and PDSI groups were found only for left‐hemisphere basal ganglia seeds (Table 4), raising the possibility that the mechanisms of speech impairment in PD may arise primarily from disruption of left‐hemisphere basal ganglia connectivity.

### 4.1 Abnormal left putamen connectivity in PDSI

We confirmed the prediction that compared to OHCs the PDSI subjects would have abnormal left‐hemisphere striatal connectivity to cortical regions involved in speech production. Although we found no differences in the connectivity of left putamen to SMA or premotor cortex, our results show that left putamen connectivity with sensorimotor cortex and STG is indeed lower in PDSI relative to OHC (Figure 2). We also confirmed the prediction that when compared to PDN, PDSI subjects would have abnormal striatal connectivity to STG, but not motor cortices (Figures 2 and 3). This finding is consistent with a study by Simonyan et al. (2013) who found that BOLD activity in the left anterior putamen was positively correlated with activity in left STG during sentence production. The results of these comparisons suggest that, while PDSI subjects have widespread reductions in connectivity between the left putamen and cerebral cortex (including cortical areas involved in speech production), reduced functional connectivity between the putamen and left STG may be uniquely linked to speech impairments in PD.

It is possible that reduced connectivity of the left putamen with left STG reflects a mechanism of impaired speech error detection and correction in PD. STG serves as functional integration area with partial overlap between speech perception and production mechanisms (Price, 2012). This functional overlap makes STG uniquely suited to detect and integrate auditory feedback during speech production (Behroozmand et al., 2015, 2016; Hickok & Poeppel, 2007; Parkinson et al., 2012; Paus, Perry, Zatorre, Worsley, & Evans, 1996; Tourville & Guenther, 2011; Tourville, Reilly, & Guenther, 2008). The STG cluster identified in the present study corresponds closely to an anterolateral region of Heschl's gyrus that electrocorticography data has linked to online voice error correction following rapid perturbations in auditory feedback (Behroozmand et al., 2016). Compared to healthy individuals, those with PD respond to rapid perturbations in auditory feedback with an exaggerated compensation in vocal output compared to healthy controls (Chen et al., 2013; Huang et al., 2016; Liu, Wang, Metman, & Larson, 2012). It has thus been suggested that people with PD have impaired feedforward control of speech production and, as a result, rely more heavily on sensory feedback integration (Liu et al., 2012). Our findings raise the possibility that, in addition to impaired feedforward control, there may be impaired auditory feedback integration mediated by decreased connectivity between left putamen and left STG. For example, not only do people with PD respond to rapid auditory perturbations with exaggerated vocal responses (Chen et al., 2013; Huang et al., 2016; Liu et al., 2012), they also appear to compensate less than controls when adapting to long‐term alterations in auditory feedback (Mollaei, Shiller, & Gracco, 2013). Decreased coupling of left putamen and left STG could thus reflect difficulties in integrating sensory information during speech production in PD.

It is also interesting to note that, in addition to articulatory models of speech production such as DIVA, models of prearticulatory error monitoring suggest that STG may also utilize perceptual feedback in the detection of phonological errors (Indefrey & Levelt, 2004), which research has shown to be abnormal in PD (Gauvin et al., 2017; McNamara, Obler, Au, Durso, & Albert, 1992). This raises another possibility that our observed reductions in left putamen–left STG connectivity in PDSI could be linked to broader changes in the online detection and correction of speech errors in PD. Whether this finding is in fact related to changes in auditory‐motor integration or an even more global effect of impaired speech error monitoring can be tested in the future using direct behavioral probes of speech error detection.

### 4.2 Abnormal left GPi connectivity in PDSI

The present study also identified group differences in cortical connectivity with left GPi. Compared to the PDN group, PDSI subjects exhibited stronger functional connectivity between left GPi and three cortical regions—the left PMd/LMC, the left angular gyrus, and the right angular gyrus. However, there were no statistically significant differences when comparing either PDN or PDSI groups to older healthy controls (Figures 3 and 4). As shown in Figures 5 and 6, the means of the OHC group appear to be different from both PDN and PDSI groups, which raises the question of whether our study was adequately powered to detect the differences. The standard error of the connectivity for all three GPi connections was higher in healthy controls compared to the PDN and PDSI groups. Our sample size analysis showed that we may have been able to detect a difference with a much larger sample size. However, as we did find statistical significance for several comparisons with our OHC group, another contributory factor in failing to detect statistically significant results could be the quality of the signal in the GPi. With this in mind, it is interesting to note that rather than observing progressively increased functional connectivity from OHC to PDN to PDSI groups, we observed the lowest levels of functional connectivity in the PDN group and the highest levels of functional connectivity in the PDSI group. This same pattern was observed for each of the three left GPi connections (left PMd/LMC, left angular gyrus, and right angular gyrus). One possible explanation is that these three pathways undergo initial disease‐related decreases in functional connectivity followed by an increase in compensatory functional connectivity once speech symptoms emerge. These three cortical connections with left GPi may thus represent pathways that compensate for functional losses in speech production. As most individuals with PD will eventually develop some form of speech impairment, this could be assessed in the future by analyzing resting‐state data for the same PDN subjects once they begin to present with speech symptoms. Below, we address our findings in the context of compensatory reorganization. However, in doing so, we acknowledge that our discussion is speculative and that elevated GPi connectivity in PDSI could be related to disease pathology rather than compensation.

The discovery of increased left GPi connectivity to left PMd/LMC is particularly intriguing given that it is located on the anterior bank of the precentral gyrus. While this area falls within the functional boundaries of the dorsal premotor cortex, it also corresponds closely with the dorsolateral laryngeal motor cortex defined by Brown et al. (2009). In light of this, we discuss two interpretations of this finding based on whether this cluster is interpreted as a premotor or primary motor region. When considered as a premotor region, increased left GPi–left PMd/LMC connectivity in PDSI subjects could be related to a greater reliance on external cues to compensate for internal cueing deficits during speech production. Problems with internal cueing have been well documented in PD (Jahanshahi et al., 1995; Siegert, Harper, Cameron, & Abernethy, 2002) and are thought to play a role in PD dysarthria (Sapir, 2014). Compared to habitual (internally cued) speech, measures of speech function and intelligibility improve when PD subjects are prompted (externally cued) to speak more loudly, clearly, or slowly (Dromey & Ramig, 1998; Ho, Bradshaw, Iansek, & Alfredson, 1999; Sapir, 2014; Tjaden, Sussman, & Wilding, 2014). As motor preparatory activity in PMd is biased toward the planning and execution of movements that are externally cued (Halsband, Matsuzaka, & Tanji, 1994; Halsband & Passingham, 1982; Lu, Arai, Tsai, & Ziemann, 2012; Mushiake, Inase, & Tanji, 1991), increased connectivity with GPi could reflect a mechanism for compensatory reliance on external cues during speech production in PD.

The second interpretation considers this cluster to be a primary motor region for laryngeal control—specifically, the dorsolateral laryngeal motor cortex (Brown, Ngan, & Liotti, 2008; Brown et al., 2009). Although the dorsolateral laryngeal cortex is located within the bounds of the premotor cortex, it is considered one of two primary motor regions for voluntary vocalization in humans (Brown et al., 2008, 2009; Simonyan, 2014) and is homologous to laryngeal motor cortex in nonhuman primates (Simonyan, 2014). Voice abnormalities are prominent in PD (Logemann, Fisher, Boshes, & Blonsky, 1978; Sapir, 2014), with perceptual characteristics including reduced loudness, reduced pitch and intensity variability, harshness, and breathiness (Darley et al., 1969; Duffy, 2013). It is therefore not surprising that we observed differences in basal ganglia connectivity with laryngeal motor cortex when comparing PDSI subjects to PDN subjects. One possibility is that increased connectivity between the two structures is in fact related to the disease process, similar to the observed hyperconnectivity of the subthalamic nucleus to motor cortices in PD (Baudrexel et al., 2011; Kurani et al., 2015). However, in the context of compensatory effects, it is also possible that PDSI subjects require greater coupling between left GPi and left laryngeal motor cortex in order to overcome disease‐related changes in voice production (e.g., hypophonia). In either case, our finding that PDSI subjects have abnormal connectivity between left GPi and left PMd/LMC lays the foundation for new hypotheses about the role of GPi connectivity in voice and speech production in PD.

The prospect of a compensatory increase in connectivity between left GPi and bilateral angular gyrus in PDSI is consistent with the current literature on resting‐state connectivity in PD (Tahmasian et al., 2017). Located in the inferior parietal lobule, the angular gyrus serves as a multimodal association area, facilitating mental processes such as arithmetic (Arsalidou & Taylor, 2011), visuospatial attention (Nobre et al., 1997), memory (Kim, 2010; Spaniol et al., 2009; Vilberg & Rugg, 2008) sequence learning (Rosenthal, Roche‐Kelly, Husain, & Kennard, 2009), and semantic processing (Benson et al., 2001; Obleser, Wise, Dresner, & Scott, 2007; Price, Peelle, Bonner, Grossman, & Hamilton, 2016; Price, 2012). Further, the posterior aspect of the angular gyrus serves as part of the default mode network (DMN), which is most active during rest or fixation and becomes deactivated when performing cognitive tasks. A recent meta‐analysis of whole‐brain resting‐state connectivity in PD found converging evidence for elevated functional connectivity of bilateral angular gyrus in PD compared to healthy controls (Tahmasian et al., 2017). The authors similarly proposed that the elevated functional connectivity in PD was due to a compensatory reorganization of intrinsic resting‐state networks following the loss of dopaminergic neurons. In line with this idea is a separate meta‐analysis of task fMRI data showing that PD patients off medication have greater activity in superior and inferior parietal cortex than controls when performing externally cued (but not internally cued) motor tasks (Herz, Eickhoff, Lokkegaard, & Siebner, 2014). If stronger left GPi–angular gyrus connectivity in PDSI subjects is in fact compensatory, it could indicate that these individuals have a greater reliance on cortical regions involved in multisensory integration or higher level associative processing.

Still, given the diversity of behavioral functions supported by the angular gyrus, it is challenging to generate hypotheses about its role in PDSI based on resting‐state data alone. While it seems reasonable to suggest that our observations reflect the compensatory recruitment of bilateral angular gyrus, it is possible that these findings are related to group differences in semantic processing. The dorsal angular gyrus, which corresponds to our present findings, has been proposed as functional subdivision involved in searching for semantic information (Seghier, Fagan, & Price, 2010) and bottom‐up semantic processing (Whitney, Grossman, & Kircher, 2009). Semantic processing difficulties have been documented in PD, even in the absence of dementia or cognitive impairment (Boulenger et al., 2008; Roberts et al., 2017; Rodriguez‐Ferreiro, Menendez, Ribacoba, & Cuetos, 2009; Signorini & Volpato, 2006). As longitudinal changes in UPDRS Part III speech impairment scores have been shown to correlate with impaired semantic verbal fluency in PD (Gago et al., 2009), it is possible that elevated connectivity between left GPi and bilateral angular gyrus reflects differences in semantic processing between PDN and PDSI groups. Future studies will be needed to examine whether elevated left GPi–angular gyrus connectivity in PDSI is related to disease mechanisms, compensatory recruitment of multisensory integration cortices, or group differences in semantic processing.

### 4.3 Correlation with motor severity

As this was the first study of resting‐state basal ganglia connectivity to systematically disentangle PD speech impairment from more generalizable motor impairments, it was important to establish whether differences in PDN and PDSI groups might be related to global motor severity. Of the four resting‐state connections that differed between PDN and PDSI subjects, none were found to correlate with MDS‐UPDRS Part III scores (Figure 7). While there is likely a strong degree of overlap between the mechanisms of speech impairments and general motor impairments in PD, the findings of this study suggest that there may be additional neural processes at play that are speech specific. One might easily predict that abnormal basal ganglia connectivity to STG and angular gyrus would not be correlated with motor severity, as these regions are not directly involved in motor output. However, it is intriguing that there was also no correlation between motor severity and left GPi–left PMd/LMC connectivity, as this could be indicative of speech specific changes in motor cortices. That these connections did not correlate with our measure of motor severity suggests that group differences observed in those basal ganglia connections are indeed independent of overall motor impairment and may be specific to speech impairments in PD. However, it remains to be seen whether correlations will emerge at more advanced stages of PD.

### 4.4 Correlation with PD medication dosage

Consistent with the motor severity scores, the connectivity of left putamen–left STG, left GPi–left angular gyrus, and left GPI–right angular gyrus were not correlated with LEDD. However, the functional connectivity between left GPi and left PMd/LMC was inversely correlated with LEDD within the PDN group alone (Figure 8). This finding is in line with prior work showing that levodopa can reduce striatal hyperconnectivity with motor cortices in PD (Kwak et al., 2010). It also suggests that antiparkinsonian medication reduces connectivity between left GPi and left PMd/LMC in PDN subjects, but not in PDSI subjects. Although levodopa provides effective treatment for motor symptoms in the early to moderate disease stages (Jankovic & Aguilar, 2008), the effect of levodopa on speech production is less consistent (Pinto et al., 2004; Schulz & Grant, 2000). If we consider hyperconnectivity of left GPi and left PMd/LMC to be a disease‐related phenomenon, it is possible that this pathological increase in connectivity contributes to speech impairments in PDSI subjects and is not responsive to levodopa in these individuals. Alternatively, if we consider hyperconnectivity to be a compensatory phenomenon, those who are levodopa responsive may no longer have a need for increased coupling between left GPi and left PMd/LMC due to treatment effects elsewhere in the brain. Further study is needed on the effects of PD medication on left GPi–left PMd/LMC connectivity during speech production. Assessment of basal ganglia connectivity on both OFF and ON medication states will provide additional insight into the differential effect of levodopa on PDN and PDSI individuals.

### 4.5 Lateralization effects

It is interesting to note that differences between PDN and PDSI groups were found only for left‐hemisphere basal ganglia seeds. Given that the cortical representation of speech and language is predominantly left‐sided and that speech production involves the left‐lateralized dopamine release in the striatum (Simonyan et al., 2013), it is not surprising that speech impairments in PD would be linked to changes in left‐hemisphere basal ganglia function. The hemisphere‐specific findings of the present study may correspond to differences in disease lateralization between the two groups. While the PDN group had an equivalent number of subjects with left‐lateralized versus right‐lateralized motor symptoms, nearly 75% of the PDSI group had symptoms that were right‐lateralized (Table 2), indicating degeneration of left‐hemisphere basal ganglia pathways. It is possible that earlier dopamine depletion in left‐hemisphere basal ganglia pathways causes PD patients with right‐lateralized motor symptoms to develop speech impairments earlier in the disease process compared to those with left‐lateralized symptoms. However, further research into speech function and disease lateralization is required before any firm conclusions can be made. If confirmed, our left‐lateralized findings could provide insight into previously observed treatment‐related shifts in cortical activity from the left to right hemisphere following successful speech therapy in PD (Narayana et al., 2010). Future work could address the hypothesis that speech impairments in PD arise primarily from changes in left cortico‐basal ganglia pathways and that treatment facilitates a functional shift of cortical activity to the right hemisphere. While intriguing, support for this hypothesis is tempered by the fact that speech impairment in PD has also been linked to reduced functional connectivity of right striatal seeds when comparing PD subjects to healthy controls (Elfmarkova et al., 2016; New et al., 2015). However, as previously mentioned, these right‐lateralized findings involved comparing a single heterogeneous group of PD subjects (including those with and without speech impairment) to OHCs. Therefore, it may be the case that while the disease impacts both left and right striatal seeds, PD patients with speech impairment experience significantly greater changes in left‐hemisphere basal ganglia function.

### 4.6 Limitations and future directions

The current study provides new insights into the roles of left putamen and left GPi in PD speech impairment; however, there are a few limitations to address. First, sample size of our OHC group was relatively small compared to the sample sizes of our PDN and PDSI groups. This is due to the smaller pool of resting‐state scans available from the PPMI Control Cohort (*n* = 21) compared to the PD Cohort (*n* = 90), which resulted in a smaller sample size once our inclusion criteria were applied (OHC: *n* = 12; PDN: *n* = 42; PDSI: *n* = 35). As a result, we may have had insufficient power to detect more subtle differences between the PD and OHC groups. Second, the MDS‐UPDRS Part III Speech Impairment score is a course, subjective measure of overall speech function in PD and cannot provide fine‐grained information about the nature of the speech impairment (i.e., articulation, voice, prosody etc.). Future studies will focus on collecting prospective fMRI data alongside acoustic and perceptual measures of speech in order to link abnormal basal ganglia connectivity with specific speech symptoms in PD. Moving forward, it will be important to conduct task‐based connectivity analysis of putamen and GPi seeds to confirm whether these connections are in fact functioning abnormally during active speech production. By corroborating our findings in both resting‐state and task fMRI, we will be able to establish a more complete understanding of the role that functional basal ganglia connections play in the emergence of speech impairments in PD.

## 5 CONCLUSION

The present study demonstrates that there are distinct functional connections between the basal ganglia and cortex that differentiate PD patients with and without speech impairment. These findings point to abnormal resting‐state connectivity of left putamen–left STG, left GPi–left PMd/LMC, left GPi–left angular gyrus, and left GPi–right angular gyrus connections as potential mechanisms for speech impairment in PD.

## CONFLICT OF INTEREST

The authors of this manuscript have no conflicts of interest to declare.
